# Supplementary material for: Endemic Melioidosis in Southern China: Past and Present
Source: Trop Med Infect Dis. 2019 Feb 25;4(1):39. doi: 10.3390/tropicalmed4010039 (PMC6473618; doi:10.3390/tropicalmed4010039)
Supplement: Supplementary file 1 [file tropicalmed-04-00039-s001.pdf]

Supplementary Table S1. Summary of the number of human melioidosis cases from endemic and non-endemic provinces of mainland China identified from different sources.

Province	ICDC Surveillance(n)	MLST Database(n)	Literature Review(n)	Total (n)
Hainan	392	191	832[1-17]	401[1]
Guangdong	1[18]	0	46[19,20]	47
Guangxi	3	0	7[21]	10
Fujian	0	0	1[22]	1
Other provinces	0	1[2]	9[23-31]	10

[1] There are numerous duplicate reports of cases in the literature, thus we adopted a relatively conservative estimate of human melioidosis comprising those from ICDC surveillance (392 cases between 2002-2016) and the cases documented prior to 2002 within the MLST database[1] (1) and from the literature[2] (8).

[2] The case had a history of residence in Malaysia before onset of the disease and was diagnosed at Chengdu, Sichuan province in 2011.

References

1. Li, L.; He, Y.W., *Pseudomonas pseudomallei* and melioidosis in China. *Chin Med J*. **1992**, 105(9), 775-779.
2. Yang, S.; Tong, S.; Mo, C.; Jiang, Z.; Yang, S.; Ma, Y.; Lu, Z., Prevalence of human melioidosis on Hainan island in China. *Microbiol Immunol* **1998**, 42(9), 651-654.
3. Lin, R.; Chen, H.; Yun, W., Clinical analysis of 12 cases of melioidosis in Hainan Province, 2002-2005. *Chin J Epidemiol* **2005**,26 (10), 824.
4. Cai, D.; Jia, J.; Wu, T.; Su, L. Clinical analysis of 32 *Pseudomonas pseudomallei* pneumonia patients. *China Tropical Medicine* **2006**,6(4), 620-621.
5. Quan, H.; Fu, H.; Mo, C., Clinical analysis of 19 cases of type 2 diabetes mellitus complicated with septicemic melioidosis. *Chin J Endocrinol Metab* **2008**, 24(1), 66-67. DOI:10.3321/j.issn:1000-6699.2008.01.020.
6. He, X.; Jia, J., Clinical analysis of 25 cases of septicemic melioidosis. *China Journal of Modern Medicine* **2008**, 18(7), 956-958.
7. Cai, D.; Jia, J., Clinical features and drug resistance analysis of 104 melioidosis cases from Hainan. *Chongqing Medicine* **2009**, 38(18), 2362-2363.
8. Lin, R.; Xie, C.; Chen, H.; Huang, Y., Clinical features and drug resistance analysis of melioidosis: 122 cases from Hainan. *Guangdong Medicine* **2011**, 32(17), 2303-2304.
9. Wu, H.; Wang, X.; Huang, D., Laboratory culture and identification on a collection of 95 *Burkholderia pseudomallei* strains. *Chinese Journal of Zoonoses* **2013**, 29(7), 730-732.
10. Zhong, Y.; Lin, H., Clinical features and follow-up of melioidosis: 40 cases in Hainan island. *Chinese Journal of Lung Disease* **2014**, 7(2), 55-57.
11. Zhong, J.; Chen, R., Clinical analysis of 40 patients infected with *Burkholderia pseudomallei* in Sanya City. *China Tropical Medicine* **2014**, 14(9), 1147-1149.
12. Fang, Y.; Chen, H.; Li, Y.L.; Li, Q.; Ye, Z.J.; Mao, X.H., Melioidosis in Hainan, China: A restrospective study. *Trans R Soc Trop Med Hyg* **2015**, 109(10), 636-642.
13. Wang, X.M.; Zheng, X.; Wu, H.; Zhou, X.J.; Kuang, H.H.; Guo, H.L.; Xu, K.; Li, T.J.; Liu, L.L.; Li, W., Multilocus sequence typing of clinical isolates of *Burkholderia pseudomallei* collected in Hainan, a tropical island of southern China. *Am J Trop Med Hyg* **2016**, 95(4), 760-764.
14. Zheng, X.; Wang, L.; Zhu, X.; Chen, H.;, Wu, H.; Mai, W.; Li, W.; Xia, L., Preliminary study on incidence of melioidosis and molecular characteristics of clinical strains of *Burkholderia pseudomallei* after Typhoon Rammasun attack. *Dis Surveill* **2016**, 31(8), 628-632. DOI: 10.3784/j.issn.1003-9961.2016.08.004
15. Zhan, Y.; Wu, Y.; Li, Q.; Yu, A., Neuromelioidosis: A series of seven cases in Hainan province, China. *J Int Med Res* **2017**, 45(2), 856-867.

16. Dong, S.; Lin, X.; Fu, S.; Wu, Q.; Xia, Q., Epidemiological features and clinical manifestations of melioidosis in 46 patients in Hainan. *Journal of pathogen biology* **2017**, 12(6), 579-582.
17. Zou, W.; Li, J.; Deng, C.; Liu, L.; Li, S., Clinical features of 35 cases of melioidosis. *Chinese Journal of Infection Control* **2018**, 17(2), 146-150.
18. Li, X.Y.; Ke, B.X.; Chen, C.N.; Xiao, H.L.; Liu, M.Z.; Xiong, Y.C.; Bai, R.; Chen, J.D.; Ke, C.W., First co-infection case of melioidosis and Japanese encephalitis in China. *BMC Infect Dis* **2018**, 18(1), 452.
19. Chen, G.; Zeng, X.; Feng, X.; Liang, T.; Ke, S., Analysis of clinical feature and epidemiology investigation of melioidosis in Leizhou peninsula of Guangdong. *Chin J Infect Dis* **2006**,24 (06), 406-409.
20. Tan, G.; Li, J.; Chen, L.; Chen, X.; Zhang, S.; Ke, L.; Liu, J., Prostatic melioidosis rarely reported in China: Two cases report and literatures review. *International journal of clinical and experimental medicine* **2015**, 8(11), 21830-21832.
21. Tang, Y.; Deng, J.; Zhang, J.; Zhong, X.; Qiu, Y.; Zhang, H.; Xu, H., Epidemiological and clinical features of melioidosis: A report of seven cases from southern inland China. *Am J Trop Med Hyg* **2018**, 98(5), 1296-1299.
22. One rare case of severe pneumonia caused by melioidosis infection from Putian [in Chinese]. Sina Fujian, 27 November 2014. Available online: http://fj.sina.com.cn/news/s/2014-11-27/detail-iavxeafr5320356.shtml (accessed on 10 Dec 2018).
23. Tu, B.; Qin, E.; Zhao, M., A case of diabetes complicated with melioidosis septicemia. *Journal of Clinical Research* **2011**, 28(11), 2224-2224.
24. Jin, H.; Chen, X.; Pan, Y.; Zhou, C.; Gao, X.; Hu, B.; He, L., Analysis and appraisal of difficult cases: No. 76, *Burkholderia pseudomallei* infection with fever, cough, swelling and pain of the right knee joint and bilateral pleural effusion with left hydropneumothorax. *National Medical Journal of China* **2005**,85(18), 1287-1289. DOI: 10.3760/j:issn:0376-2491.2005.18.021
25. Liu, Y.; Zhou, W.; Zhang, Z.; Si, J., One case of septicemia caused by *Burkholderia pseudomallei*. *Laboratory Medicine and Clinic* **2016**, 13(4), 575-576. DOI: 10.3969/j.issn.1672-9455.2016.04.061
26. Fang, Y.; Chen, J.; Zhu Y.; Mao,X., Source tracking for an imported case of melioidosis. *J Third Mil Med Univ* **2016**, 38(11), 1224-1225.
27. Li, Y.; Zhong, Y.; Liu, W.; Pu, Y.; Li, H.; Yan, Q.; Zou M.; Guo, S., One death of sepsis caused by *Burkholderia pseudomallei*. *Chinese Journal of Infection Control* **2013**, 12(6), 475-476. DOI: 10.3969/ j.issn.1671- 9638.2013.06.025
28. Zhang, H., First imported and death case of melioidosis in Qinghai Province, China. *International Journal of Laboratory Medicine* **2015**, 36(12), 1798-1799. DOI: 10.3969/j.issn.1673-4130.2015.12.075
29. Yang, L.; Lu, J., A case of cutaneous melioidosis infection misdiagnosed as rash over 10 years. *J Third Mil Med Univ* **2014**, 36(6), 552-552.
30. Wang, Y., A case of melioidosis. *Journal of Clinical Dermatology* **2014**, 43(3), 163-164.
31. Li, L.; Wang, C.; Liu, J.; Gu, X.; Liang, R.; Sun, H., A case of *Burkholderia pseudomallei* septicemia associated with periorbit abscess. *Chinese Journal of Infection Control* **2017**, 16(6), 574-576. DOI:10.3969/ j.issn.1671-9638.2017.06.021.
